# Some insights on traditional and novel approaches in microbial biotechnology that contribute to the United Nations Sustainable Development Goals

**DOI:** 10.1111/1751-7915.14318

**Published:** 2023-07-15

**Authors:** Gloria Soberón‐Chávez

**Affiliations:** ^1^ Departamento de Biología Molecular y Biotecnología Instituto de Investigaciones Biomédicas, Universidad Nacional Autónoma de México, Ciudad Universitaria Ciudad de México Mexico

The 17 sustainable developmental goals (SDGs) established by the United Nations (UN) General assembly in 2015 are the following:

1. No poverty.
2. Zero hunger.
3. Good health and well‐being.
4. Quality education.
5. Gender equality
6. Clean water and sanitation.
7. Affordable and clean energy.
8. Decent work and economic growth.
9. Industry, innovation, and infrastructure.
10. Reduced inequalities.
11. Sustainable cities and communities.
12. Responsible consumption and production.
13. Climate action.
14. Life below water.
15. Life on land.
16. Peace, justice and strong institutions.
17. Partnership for the goals.

All these goals are interconnected, and there are many ways that microbial technologies have impacted each of them. Therefore, the examples of microbial technologies that may impact SDG that I include in this ‘Burning Questions’ editorial do not wish to be exhaustive, but to highlight the enormous potential of microbial biotechnology. Furthermore, recently an editorial was published which addressed how microbial technologies have contributed to the UN SDG and thus have played an important role in diminishing the risk of social conflicts; how microbial technologies are important to reach peace (Anand et al., 2023). This ‘Burning questions’ editorial aims to complement some of the microbial technologies that were described in the Anand et al. (2023), contribution, and to reinforce the importance of using microbes and their products for getting closer to reaching UN SDG in the near future.

SDG 2 is about achieving a world free of hunger by 2030. Microbial biotechnology has traditionally been linked to food technologies, in different aspects. In this respect, first I wish to highlight some aspects of traditional fermented food and their potential industrialization. Food fermentation is the most economical method to produce and preserve food, and it is the most ancient method that all cultures have used (Rizo et al., 2020). In addition, traditional fermented foods are crucial for maintaining biodiversity of supplies since nine crops account for 66% of the total crop production (State of the World's Biodiversity for Food and Agriculture FAO, February 22, 2019), and they constitute high‐fibre diets which have crucial role in health and nutrition (Reynolds et al., 2020). The microbiome associated with traditional fermented foods has not been very well characterized (Rizo et al., 2020), except for highly industrialized foods such as cheese, wine, bread and beer, but recently the contribution of bacteria synthetizing extracellular polysaccharides such as fructan, that represent an important percentage of their fibre has been recognized (Vallejo‐García et al., 2023). The solution to the hunger crisis cannot be attained by westernizing the food industry, but new approaches and strategies should be used to producing locally fermented traditional foods with a higher productivity and quality standards (Hugenholtz, 2013).

Human population is increasing at a very high rate, and it increased 2.5 times from 1960 to 2020 reaching 8 billion people on November 15, 2022, so land for agriculture and farming is increasingly limited. Therefore, new strategies should be developed for food production that optimizes land productivity, without harming the environment, and new sources of nutrients should be made available.

Recently, mycoprotein has reached the marked in nearly 20 countries around the world (under the brand name of Quorn) and has been incorporated in different products. This food ingredient is rich in both insoluble fibre and protein and is produced from the fermentation of a fungus called *Fusarium venenatum* (Finnigan, 2011; Finnigan et al., 2017). In addition, research on different sources of microbial proteins for the food industry is an active field (Bajić et al., 2022) and might represent an alternative source of protein for human and animal feeding.

The intensification of agriculture is a need for producing enough food for the rapidly growing population. In this respect, I want to comment on two aspects of microbial biotechnology (for a comprehensive review consult Anand et al., 2023), the use of bioinsecticides and the approach of extending the benefits of biological nitrogen fixation for non‐legume crops such as cereals (Ladha et al., 2022).

In the last 60 years, we have witnessed a considerable increase in agriculture yield, mainly in high‐income countries, that has been called the green revolution. This technology‐intensive agriculture relies on the use of inorganic fertilizers, chemically synthetized insecticides and other supplies that are out of reach for smallholder farmers in low‐income countries, a situation that causes profound inequities. In addition, the use of fertilizers and pesticides has caused severe water and soil pollution (Baron, 1992).

An alternative for the use of chemically synthetized insecticides is the use of bioinsecticides, which include bacteria, mainly *Bacillus thuringiensis*, virus and fungi which are non‐toxic and highly specific for certain pests, but they only constitute around 2% of the insecticidal market (Soberón et al., 2023). Bioinsecticides efficacy must be improved, and the cost of their production reduced, so they can compete with synthetic insecticides and their advantage of being more environmentally friendly and less toxic can be exploited.

As stated above, highly technologized agriculture is dependent on fertilization, markedly by nitrogen. The use of nitrogen fertilizers accounts for high greenhouse emissions, water pollution and eutrophication. However, symbiotic nitrogen fixation by the *Rhizobium*‐legume interaction has been widely studied in the light of sustainable production of food (Lindström & Mousavi, 2020). These bacteria have been used to produce *Rhizobium*‐based inoculants that reduce the use of these fertilizers with legume crops. Therefore, making symbiotic nitrogen fixation available to non‐legume crops would represent a major breaking point in agriculture and in the possibility of ending hunger in the world.

Intensive research is being carried out to engineer nitrogen fixation in target crops based on the study of *Rhizobium* interaction with legumes, forming specialized root nodules where nitrogen fixation takes place. There are still some crucial points that are not fully understood, but it is a promising field with great scientific advances (Jhu & Oldroyd, 2023). In this regard, another strategy has been the isolation of bacteria that colonize the roots of cereals, but do not fix nitrogen and to engineer them to express nitrogen fixation genes (Ryu et al., 2020).

Transgenic plants have been developed and are commercialized by global companies, which express *B*. *thuringiensis* toxins and thus produce bioinsecticides (Soberón et al., 2023), and there is an active field of research trying to express nitrogen fixation genes in plants (Ladha et al., 2022, Li & Chen, 2020). However, transgenic plants are designed to be used in highly technologized agriculture, which as stated, is not accessible for low‐income populations. In contrast, microbial‐based technology could be used by small farmers in low‐income countries if the challenge of developing low‐cost biopesticides and nitrogen‐fixing bacteria that can be used to inoculate other crops besides legumes is met.

The threat of pollution impacts several SDGs, specifically SDG 12 which is about ensuring sustainable consumption and production patterns. Microbial biotechnologies are at the centre of pollution treatment, and we are facing problems that have not been met during the evolution of life in our planet. This is the case of plastic pollution and the formation of microplastics that are nearly ubiquitous and affect the environment and all living creatures (Schmid et al., 2021). Microplastics today numerically dominate marine debris although human‐derived debris has been thrown to the sea for millenniums. These microplastics are colonized by microbes, and this new‐to‐the‐world ecosystem has been called ‘plastisphere’ (Amaral‐Zettler et al., 2020). The study of microbes forming the plastisphere will reveal new organisms and metabolic routes that will impact the development of microbial technologies not only for plastic degradation (Rüthi et al., 2023) but might also lead to the discovery of new bioactive compounds, like biosurfactants, that might impact other areas of microbial biotechnology.

Biosurfactants comprise compounds produced by diverse types of microorganisms like bacteria and fungi, which, even having different chemical structures have the common ability to act as surfactants, having an amphiphilic nature (Baccile & Poirier, 2023). At present, two glycolipid biosurfactants have reached the market, rhamnolipids produced by *Pseudomonas* and sophorolipids produced by the yeast *Starmerella bombicola* (Zibek & Soberón‐Chávez, 2022). These tension‐active compounds have a wide range of industrial applications (Soberón‐Chávez et al., 2023), going from biomedicine to oil recovery (Nikolova & Gutierrez, 2021) and are useful for the clean‐up of soils contaminated with hydrocarbons and heavy metals (Hogan et al., 2023). The advantage of biosurfactants over chemically synthetized surfactants that are widely used in practically all industrial fields is that they are non‐toxic and biodegradable and show unexpected activities that are useful in many biotechnology areas (Kossmann et al., 2023, for example). Thus, the use of these bioactive compounds instead of their chemically synthetized counterparts will considerably reduce pollution and will benefit different industrial areas. However, to compete in the market with the chemically synthetized surfactants their production cost should be greatly reduced.

Summing up, in my opinion microbial technologies have a great potential to contribute to reaching UN SDG in several areas as has been reviewed recently (Anand et al., 2023), but we have a big challenge as a scientific community. It is important not only that we develop new microbial technologies that are useful to solve the problems faced by humanity in reaching the SDG but also that the solutions that we give can be applied locally by different communities, especially of the less favoured people. Furthermore, an important task of our community is to sensibilize policymakers and decision‐takers of the importance of microbial technologies and to insist in the importance of including microbiology and its applications as part of children education.

## AUTHOR CONTRIBUTION

The conceptualization and writing is responsibility of GSCh.

## FUNDING INFORMATION

No funding information provided.

## CONFLICT OF INTEREST STATEMENT

The author declares that she does not have any conflict of interest.
